# Combined Administration of Pravastatin and Metformin Attenuates Acute Radiation-Induced Intestinal Injury in Mouse and Minipig Models

**DOI:** 10.3390/ijms232314827

**Published:** 2022-11-27

**Authors:** Jung Moon Kim, Hyewon Kim, Su Hyun Oh, Won Il Jang, Seung Bum Lee, Mineon Park, Soyeon Kim, Sunhoo Park, Sehwan Shim, Hyosun Jang

**Affiliations:** 1 Laboratory of Radiation Exposure & Therapeutics, National Radiation Emergency Medical Center, Korea Institute of Radiological and Medical Science, Seoul 01812, Republic of Korea; 2 Department of Veterinary Surgery, College of Veterinary Medicine, Konkuk University, Seoul 05029, Republic of Korea

**Keywords:** pravastatin, metformin, radiation-induced intestinal injury, minipigs, epithelial regeneration, inflammation

## Abstract

Radiation-induced gastrointestinal (GI) damage is one of the critical factors that serve as basis for the lethality of nuclear accidents or terrorism. Further, there are no Food and Drug Administration-approved agents available to mitigate radiation-induced intestinal injury. Although pravastatin (PS) has been shown to exhibit anti-inflammatory and epithelial reconstructive effects following radiation exposure using mouse and minipig models, the treatment failed to improve the survival rate of high-dose irradiated intestinal injury. Moreover, we previously found that metformin (MF), a common drug used for treating type 2 diabetes mellitus, has a mitigating effect on radiation-induced enteropathy by promoting stem cell properties. In this study, we investigated whether the combined administration of PS and MF could achieve therapeutic effects on acute radiation-induced intestinal injury in mouse and minipig models. We found that the combined treatment markedly increased the survival rate and attenuated histological damage in a radiation-induced intestinal injury mouse model, in addition to epithelial barrier recovery, anti-inflammatory effects, and improved epithelial proliferation with stem cell properties. Furthermore, in minipig models, combined treatment with PS and MF ameliorates gross pathological damage in abdominal organs and attenuated radiation-induced intestinal histological damage. Therefore, the combination of PS and MF effectively alleviated radiation-induced intestinal injury in the mouse and minipig models. We believe that the combined use of PS and MF is a promising therapeutic approach for treating radiation-induced intestinal injury.

## 1. Introduction

Radiotherapy, mainly used to treat cancer, induces radiation-induced gastrointestinal (GI) damage, and presents a major limitation in the treatment of abdominal and pelvic cancers. It can cause GI symptoms that affect the quality of life, including abdominal pain, anorexia, vomiting, diarrhea, weight loss, and dehydration [1,2]. Furthermore, it is predicted to be a mandatory factor for survival in the event of nuclear accidents or radiological terrorism [3,4]. GI damage in acute radiation syndrome is characterized by nausea, diarrhea, and endotoxemia. Delayed radiation damage leads to chronic inflammation and fibrotic changes [5,6]. Although various therapeutic agents have been reported to minimize these effects on healthy tissues, practical medication options are lacking. Therefore, it is necessary to develop a suitable mitigating agent for radiation-induced intestinal injuries.

Statins inhibit 3-hydroxy-3-methyl-glutaryl coenzyme A reductase and are widely used as lipid-controlling drugs [7,8]. The beneficial effects of statins include modulation of the immune system, reduction of vascular inflammation with improved endothelial dysfunction, and inhibition of oxidative stress [8]. Statins exhibit a therapeutic effect on radiation-induced damage, and various studies have demonstrated their anti-inflammatory effect by regulating endothelial function after ionizing radiation [9,10,11]. In particular, pravastatin (PS) has therapeutic effects on radiation-induced intestinal damage [10,12,13,14]. The anti-inflammatory, anti-fibrotic, and barrier reconstructive effects of PS following radiation exposure have been revealed using mouse and minipig models [10,13,14]. A clinical study also showed that statin treatment was associated with a reduction in acute GI syndrome following pelvic radiotherapy [15]. Although the above-mentioned outcomes proved certain effects of PS on the prevention and treatment of radiation-induced enteropathy, it is noteworthy that radiation damage is triggered by multiple complicated crosstalk mechanisms. Thus, a single therapeutic strategy may fail; for instance, PS treatment failed to improve the survival rate of high-dose irradiated enteropathy.

Metformin (MF), a biguanide derivative, is the most extensively used drug for the treatment of type 2 diabetes mellitus. It not only lowers blood glucose levels but also has notable properties such as anti-oxidative and anti-inflammatory effects, which have been demonstrated in several studies [16,17,18]. Recent studies have shown that MF can protect healthy tissues from radiation-induced damage [17,19]. Furthermore, MF can stimulate the restoration of damaged DNA by upregulating AMP-activated protein kinase [20]. Although many studies have confirmed that MF is effective for radiation-induced injury, most studies have focused on protective purposes rather than the mitigating effects. Nevertheless, in our previous study, we found that MF mitigated radiation-induced enteropathy by promoting stem cell properties [21].

Although many studies have been conducted using mice and rats in the medical industry, several anatomical and physiological differences between humans and rodents exist [22]. Over a long period of time, minipigs have evolved from little-known alternative animal models compared with dogs and primates to promising models in the toxicology and drug development fields [23]. The minipig model mimics the human GI tract anatomy and physiology (e.g., transit time, pH value, and microbiota) [24]. Therefore, minipigs are an appropriate animal model in evaluating oral bioavailability [25]. It is a potentially more useful animal model than are rodent models in evaluating radiation-induced GI damage. As the body thickness of a minipig resembles that of a young human, the radiation absorption patterns of minipigs are similar to those of humans [26]. Therefore, the minipig model is a useful animal model for clinical therapeutic studies on radiation-induced GI damage [14,27].

A single therapy with PS or MF did not show survival effects in lethal dose irradiation. Surprisingly, the combined treatment of PS and MF notably increased the survival rate in the lethal dose-irradiated mouse model. In the minipig model, combined treatment with PS and MF improved gross pathological damage in abdominal organs and attenuated radiation-induced intestinal histological damage. Collectively, the results indicated that the combined administration of PS and MF in mouse and minipig models achieved superior therapeutic effects on acute radiation-induced intestinal injury compared to a single treatment with PS or MF.

## 2. Results

### 2.1. Effects of Combined PS and MF Treatment on Survival Rate and Radiation-Induced Intestinal Injury in the Mouse Model

**Figure 1:**
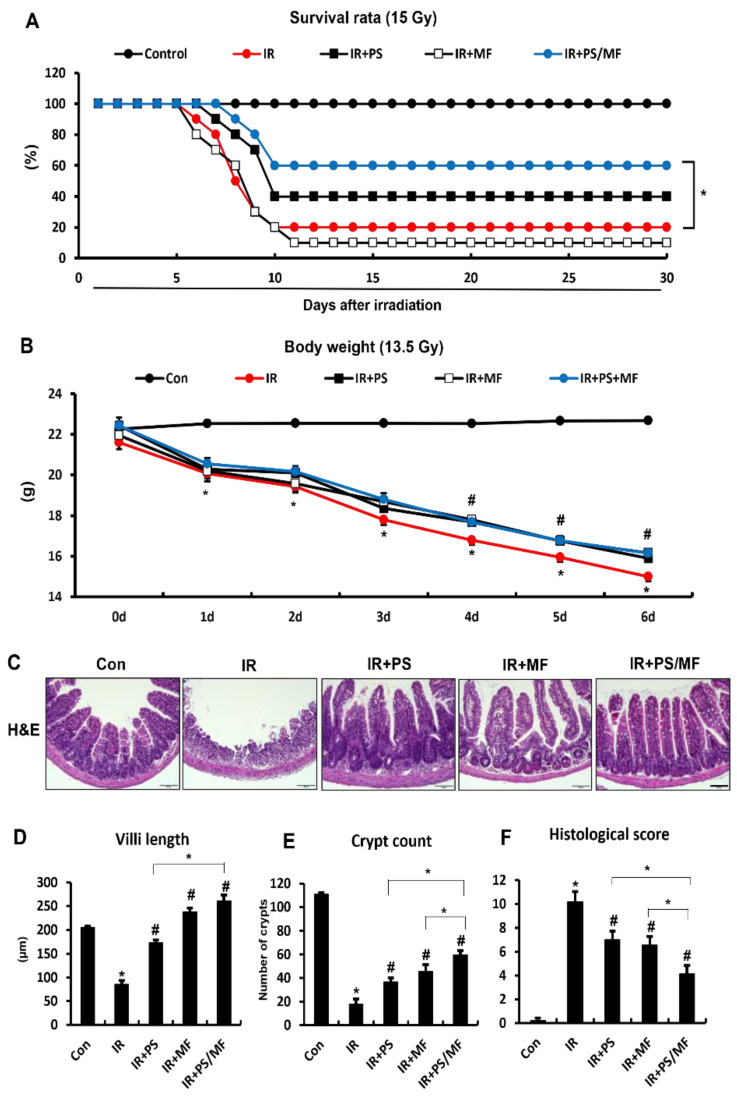
Effects of combined pravastatin and metformin treatment on survival rate and radiation-induced intestinal injury in the mouse model. (**A**) Survival Rate of control (Con), irradiated (IR), pravastatin-treated IR (IR + PS), metformin-treated IR (IR + MF), combined PS and MF-treated IR (IR + PS/MF) mice after abdominal irradiation of lethal dose of 15Gy. n = 10 mice per group (**B**) Body weights of Con, IR, IR + PS, IR + MF, IR + PS/MF groups after abdominal radiation of sub-lethal dose of 13.5 Gy. (**C**) Hematoxylin & Eosin (H&E) stain, (**D**) villi length, and (**E**) crypt count of 13.5 Gy irradiated small intestine after 6 days. Bar = 100 μm. (**F**) Histological score defined by the degree of maintenance of epithelial architecture, crypt damage, vascular dilation, and infiltration of inflammatory cells in the lamina propria (0 = none, 1 = mild, 2 = moderate, 3 = high) of small intestine from Con, IR, IR + PS, IR + MF, IR + PS/MF groups. Data are presented as the mean ± standard error of the mean; n = 5–6 mice per group. * *p* < 0.05 compared to the control; # *p* < 0.05 compared to the IR group.

To examine the effects of the combination of PS and MF on the outcome of radiation-induced intestinal injury, we performed focal irradiation of the mouse abdomen using Xrad-320 with a lethal dose of 15 Gy for survival analysis and a sublethal dose of 13.5 Gy for the therapeutic effects of radiation-induced intestinal injury. As shown in Figure 1A, MF treatment did not show a significant difference in the survival rate compared with the IR group; however, PS treatment slightly, but not significantly, increased the survival rate compared with the IR group (Figure 1A). In particular, the combination of PS and MF significantly improved the survival rate compared to the IR group (Figure 1A). In sub-lethal dose irradiation, the groups of PS, MF, and the combination of PS and MF treatment showed significantly increased body weight compared with the IR group (Figure 1B). There were no significant differences in body weight between the IR + PS, IR + MF, and IR + PS/MF groups (Figure 1B). Six days after irradiation, histological evaluation was performed to demonstrate the therapeutic effects of the combination of PS and MF on radiation-induced intestinal injury. hematoxylin and eosin (H&E) staining of the small intestine showed crypt and villus destruction, loss of epithelial layers, inflammatory cell accumulation in the mucosa and submucosa, and thickness of the submucosa in the IR group. While this histological damage was partially attenuated in the PS- or MF-treated group, the combination treatment of PS and MF improved the histological damage (Figure 1C). Villi lengths and crypt numbers were increased in the IR + PS, IR + MF, and IR + PS/MF groups compared with those in the IR group. In particular, the combination treatment with PS and MF improved the most in the groups (Figure 1D,E). Further, we evaluated the histological scores, including epithelial layer destruction, crypt damage, vascular dilation, and inflammation. The combination treatment of PS and MF significantly decreased the histological scores compared to the single treatment with PS or MF (Figure 1F). These results indicate that the combination treatment of PS and MF improved the survival rate under lethal dose radiation exposure and effectively alleviated radiation-induced intestinal injury.

### 2.2. Effect of Combined PS and MF Treatment on Epithelial Barrier Damage and Inflammatory Response in Radiation-Induced Intestinal Injury

**Figure 2:**
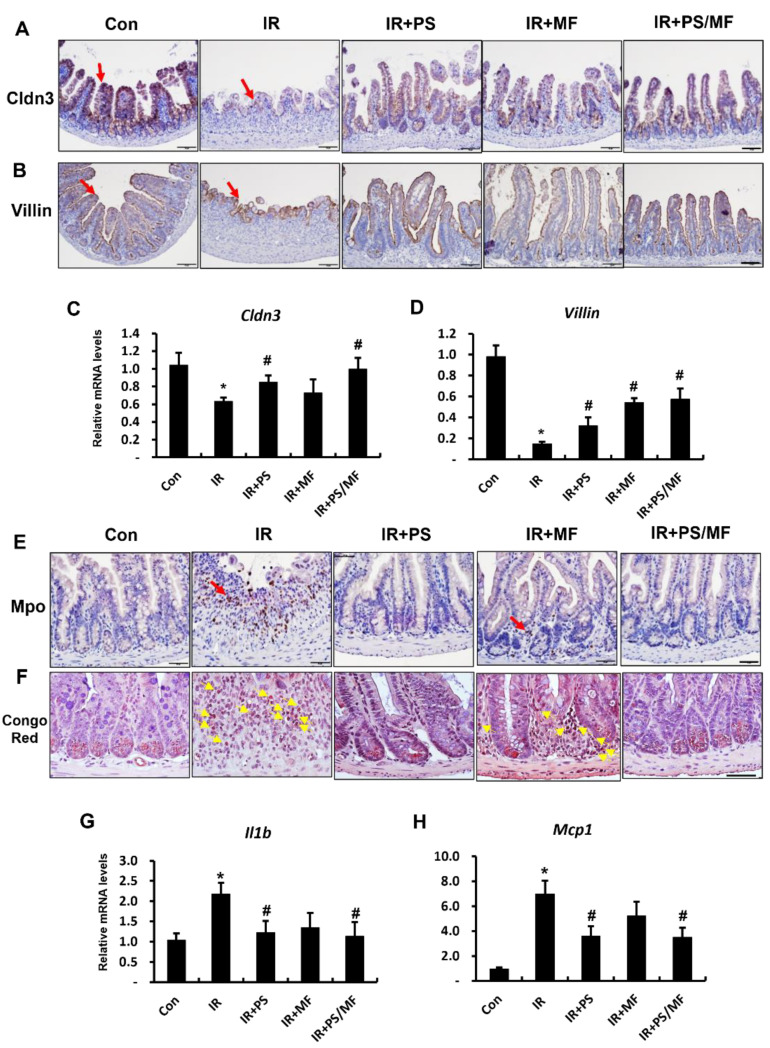
Effect of combined pravastatin and metformin treatment on epithelial barrier damage and inflammatory response in radiation-induced intestinal injury. Immunohistochemical analysis of (**A**) claudin 3 (Cldn3) and (**B**) villin in intestine tissue of control (Con), irradiated (IR), pravastatin-treated IR (IR + PS), metformin-treated IR (IR + MF), combined PS and MF-treated IR (IR + PS/MF) groups. Red arrows indicate Cldn3 and villin positive cells. Bar = 100 μm. mRNA levels of (**C**) Cldn3 and (**D**) villin of the intestine from Con, IR, IR + PS, IR + MF, IR + PS/MF groups. (**E**) Immunohistochemical analysis of myeloperoxidase (MPO) and (**F**) congo red stain for eosinophils. Red arrows indicate MPO positive cells. Yellow arrows indicate eosinophils. Bar = 50 μm. mRNA levels of (**G**) interleukin 1b (Il-1b) and (**H**) monocyte chemoattractant protein 1 (Mcp1) of the intestine from Con, IR, IR + PS, IR + MF, IR + PS/MF groups. Data are presented as the mean ± standard error of the mean; n = 5–6 mice per group. * *p* < 0.05 compared to the control; # *p* < 0.05 compared to the IR group.

Epithelial tight junctions are physical components of epithelial integrity that preserve intestinal homeostasis by controlling paracellular permeability [28,29]. In particular, claudin3 (CLDN3) is highly sensitive to radiation exposure, and rapid disruption of CLDN3 induces loss of epithelial integrity in the irradiated intestine [27,30]. Previously, we identified that PS improves radiation-induced barrier destruction by regulating intercellular junction molecules, including tight junctions [14]. The immunostaining of Cldn3 was also strongly expressed in the epithelium of the small intestinal tissue under the PS, MF, and combination treatment in PS and MF groups (Figure 2A). Moreover, we identified that mRNA levels of Cldn3 significantly increased in the IR + PS and IR + PS/MF groups than in the IR group (Figure 2C). There were no marked differences between IR + MF and IR groups. As villin is an enterocyte marker involved in the regulation of epithelial integrity, we evaluated villin expression. Villin protein was broadly expressed in the enterocytes of the IR + PS, IR + MF, and IR + PS/MF groups (Figure 2B). Additionally, mRNA levels of villin were significantly higher in the IR + PS, IR + MF, and IR + PS/MF groups than in the IR group (Figure 2D).

Radiation-induced intestinal injury is characterized by an inflammatory response, with increased leukocyte infiltration and (pro-)inflammatory cytokine expression. We examined the anti-inflammatory effects of the combined PS and MF treatment on the irradiated intestines. The increased expression of myeloperoxidase (MPO), a neutrophil marker, corresponds to the severity of inflammation in intestinal diseases. Eosinophils also play a critical role in intestinal inflammation and radiation-induced intestinal damage by releasing cytokines and chemokines [31,32]. The number of cells positive for Mpo and eosinophils increased in the IR group, and these inflammatory cells markedly decreased in the IR + PS and IR + PS/MF groups (Figure 2E,F). In contrast, MF treatment slightly decreased the number of active neutrophils and eosinophils in the irradiated intestines (Figure 2E,F). Interleukin (IL) 1β and monocyte chemoattractant protein 1 (MCP1) are well-known pro-inflammatory cytokines that cause inflammation [12,33]. The mRNA levels of Il1β and Mcp1 were markedly increased in the irradiated intestine compared with those in the healthy intestine (Figure 2G,H). However, these pro-inflammatory cytokines (Il1β and Mcp1) mRNA levels considerably decreased in the PS-treated and combined PS and MF-treated groups compared with those of the IR group. These data suggest that the combination treatment with PS and MF restores barrier integrity with epithelial reconstruction and inhibits inflammation in radiation-induced intestinal injury.

### 2.3. Effect of Combined PS and MF Treatment on Epithelial Cell Proliferation in Radiation-Induced Intestinal Injury

**Figure 3:**
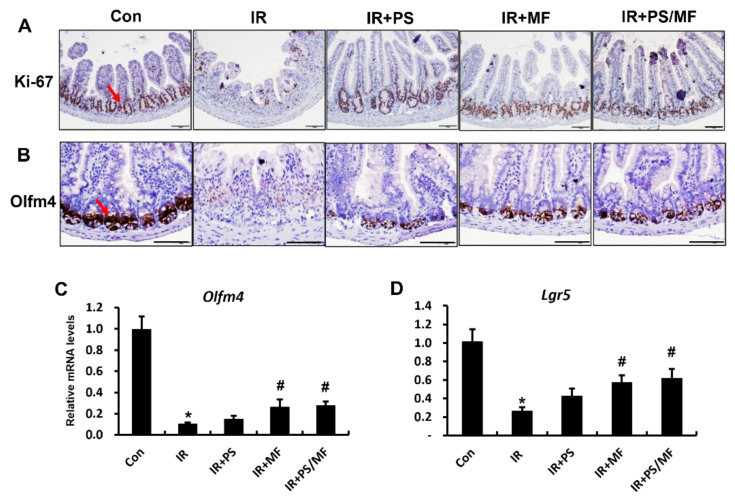
Effect of combined pravastatin and metformin treatment on epithelial cell proliferation in radiation-induced intestinal injury. Immunohistochemical analysis of (**A**) Ki-67 and (**B**) olfactomedin 4 (Olfm4) in small intestine of control (Con), irradiated (IR), pravastatin-treated IR (IR + PS), metformin-treated IR (IR + MF), combined PS and MF-treated IR (IR + PS/MF) groups. Bar = 100 μm Red arrows indicate Ki-67 and Olfm4 positive cells. mRNA levels of (**C**) Olfm4 and (**D**) leucine rich repeat containing G protein coupled receptor 5 (Lgr5) of Con, IR, IR + PS, IR + MF, IR + PS/MF groups. Data are presented as the mean ± standard error of the mean; n = 5–6 mice per group. * *p* < 0.05 compared to the control; # *p* < 0.05 compared to the IR group.

As the GI tract has a rapid self-renewal rate, intestinal stem cells are sensitive to radiation exposure. Impaired intestinal stem cells by radiation retard epithelial cell proliferation and regeneration of the intestine [34,35]. In our previous study, we found that MF treatment promoted stem cell properties and increased epithelial proliferation [21]. Therefore, we assessed the epithelial proliferation capacity and intestinal stem cell ability in the combined PS and MF treatment under radiation exposure. Ki-67 is a marker of the active state of proliferating cells and is used to identify regeneration crypts [36]. The number of Ki-67 positive cells was markedly lower in the IR group than in the control group. However, Ki-67 positive cells increased in the PS, MF, and combination of PS and MF-treated groups compared with the IR group (Figure 3A). In particular, treatment with MF alone and combined treatment with PS and MF markedly increased the Ki-67 positive cells in the irradiated intestine. The protein and mRNA expression of olfactomedin 4 (Olfm4), an active-state intestinal stem cell marker, considerably decreased in the IR group. The MF alone and the combination of PS and MF treatment markedly increased the expression of Olfm4 compared with the IR group. However, there was no significant difference in the Olfm4 mRNA levels between the IR + PS and IR groups (Figure 3C). We also evaluated another intestinal stem cell marker, leucine-rich repeat containing G protein-coupled receptor 5 (Lgr5), which is highly sensitive to radiation [37]. In the small intestine of irradiated mice, the mRNA levels of Lgr5 were notably reduced compared with those in the control group. There was a remarkable increase in Lgr5 expression in the group treated with MF alone or in combination with PS and MF. Overall, the combination of PS and MF treatment enhanced epithelial proliferation by promoting intestinal stem cell properties during radiation-induced intestinal injury.

### 2.4. Effect of Combined PS and MF Treatment on Radiation-Induced Damage in a Minipig Model: Gross Morphology of Abdominal Organs

**Figure 4:**
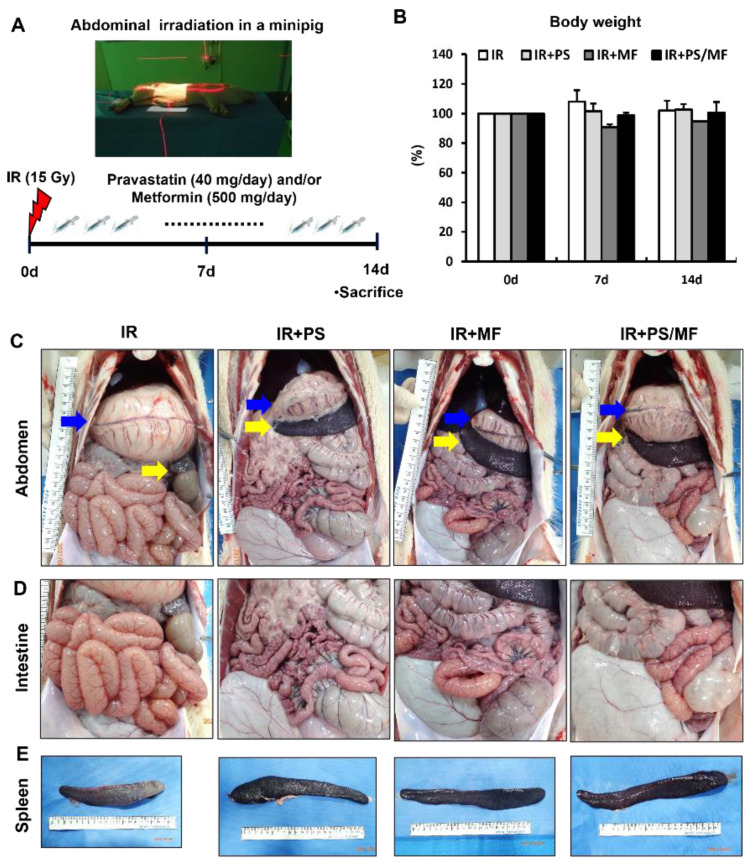
Effect of combined pravastatin and metformin treatment on radiation-induced damage in a minipig model: gross morphology of abdominal organs. (**A**) Schematic diagram of the minipig experimental protocol. Minipigs were treated with combination of pravastatin (PS) and metformin (MF) by oral administration after receiving a single dose of 15Gy gamma-ray radiation to the whole abdomen. (**B**) Body weights of minipig in irradiated (IR), PS-treated IR (IR + PS), MF-treated IR (IR + MF), combined PS and MF-treated IR (IR+PS/MF) groups after 15Gy irradiation. (**C**) Gross pathology of abdominal organs, (**D**) intestine, and (**E**) spleen at 14 days after irradiation of IR, IR + PS, IR + MF, IR + PS/MF groups. Blue arrows indicate stomach. Yellow arrows indicate spleen.

The minipig model is similar in anatomy and physiology to the human GI tract, including transit time, pH value, microbiota [24], and the radiation absorption patterns in humans [26]. Therefore, minipigs are an appropriate animal model to evaluate the effects of oral pharmaceutical treatment on radiation-induced intestinal injury. We tested whether the combination treatment of PS and MF was also effective in abdominally irradiated minipigs. After anesthetizing the minipigs, they were placed in lateral recumbency, and the abdomen was irradiated with a total dose of 15 Gy. A schematic of minipig modeling and treatment is shown in Figure 4A. After 2 weeks, there was no significant difference in body weight between the groups (Figure 4B). Necropsy was performed 2 weeks after irradiation. We investigated the gross pathology of the abdominal lesions. Abdominal irradiated minipigs showed an enlarged stomach due to poor peristalsis, spleen atrophy, thin wall, and edematous small intestine, and congested blood vessels around the intestine (Figure 4C). On the contrary, the IR + PS, IR + MF, and IR + PS/MF groups improved these pathological changes to a gross appearance close to the normal condition (Figure 4C). On gross appearance of the intestine, the IR group showed a thin and dilated intestinal wall with accumulating gas and a congested change in the vessels in the intestinal wall (Figure 4D). The other groups showed alleviation of these pathological changes (Figure 4D). In spleen morphology, atrophic changes by irradiation improved the PS, MF, and combination of PS and MF treatments (Figure 4E). Overall, the combined treatment with PS and MF alleviated the pathophysiological changes caused by radiation exposure.

### 2.5. Effect of Combined PS and MF Treatment on Histological Damage of the Intestine in the Irradiated Minipig Model

**Figure 5:**
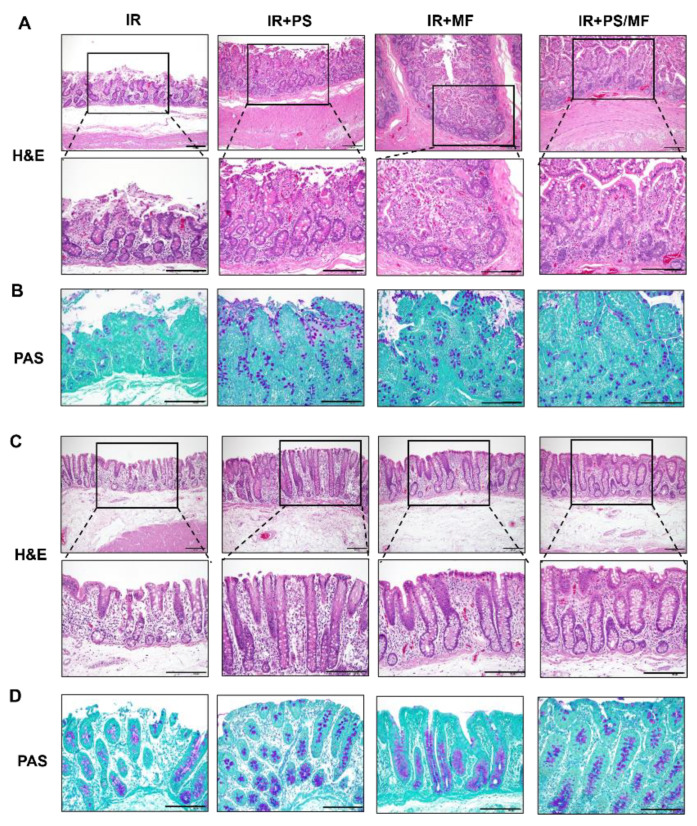
Effect of combined pravastatin and metformin treatment on histological damage of the intestine in the irradiated minipig model. (**A**) Hematoxylin & Eosin (H&E) staining of small intestinal tissues harvested from irradiated (IR), pravastatin-treated IR (IR + PS), metformin-treated IR (IR + MF), combined PS and MF-treated IR (IR + PS/MF) minipigs. Bar = 200 μm. (**B**) Periodic Acid Schiff (PAS) staining of small intestinal tissues harvested from IR, IR + PS, IR + MF, IR + PS/MF groups. Bar = 200 μm. (**C**) H&E staining and (**D**) PAS staining of large intestinal tissues harvested from IR, IR + PS, IR + MF, IR + PS/MF groups. Bar = 200 μm.

To verify the effects of combined treatment with PS and MF on radiation-induced GI injury, we performed H&E and periodic acid-Schiff (PAS) staining of intestinal tissue in a minipig model. Histological analysis showed that the irradiated small intestine displayed congested vessels, crypt loss, accumulation of inflammatory cells, a decreased number of goblet cells, and disruption of epithelial continuity (Figure 5A,B). In contrast, PS treatment improved crypt regeneration, epithelial integrity, inflammatory cell infiltration, and goblet cell maturation in the small intestine. MF treatment also recovered the loss of crypts and epithelium with goblet cells, but partial vessel congestion and inflammatory cell infiltration were observed. In particular, the combined treatment with PS and MF resulted in complete epithelial continuity and goblet cell maturation in the epithelial layers (Figure 5A,B). In the large intestine, irradiated minipigs showed severe crypt and epithelial layer loss, inflammatory cell infiltration, and goblet cell loss (Figure 5C,D). PS and MF treatment partially attenuated the histological damage caused by irradiation (Figure 5C,D). In particular, the combined treatment with PS and MF resulted in complete epithelial layers with mature goblet cells and crypt regeneration (Figure 5C,D). Overall, the combined treatment of PS and MF improves histological damage compared to monotherapy with PS or MF.

### 2.6. Effect of Combined PS and MF Treatment on Inflammatory Response and Epithelial Integrity in Irradiated Minipig Model

**Figure 6:**
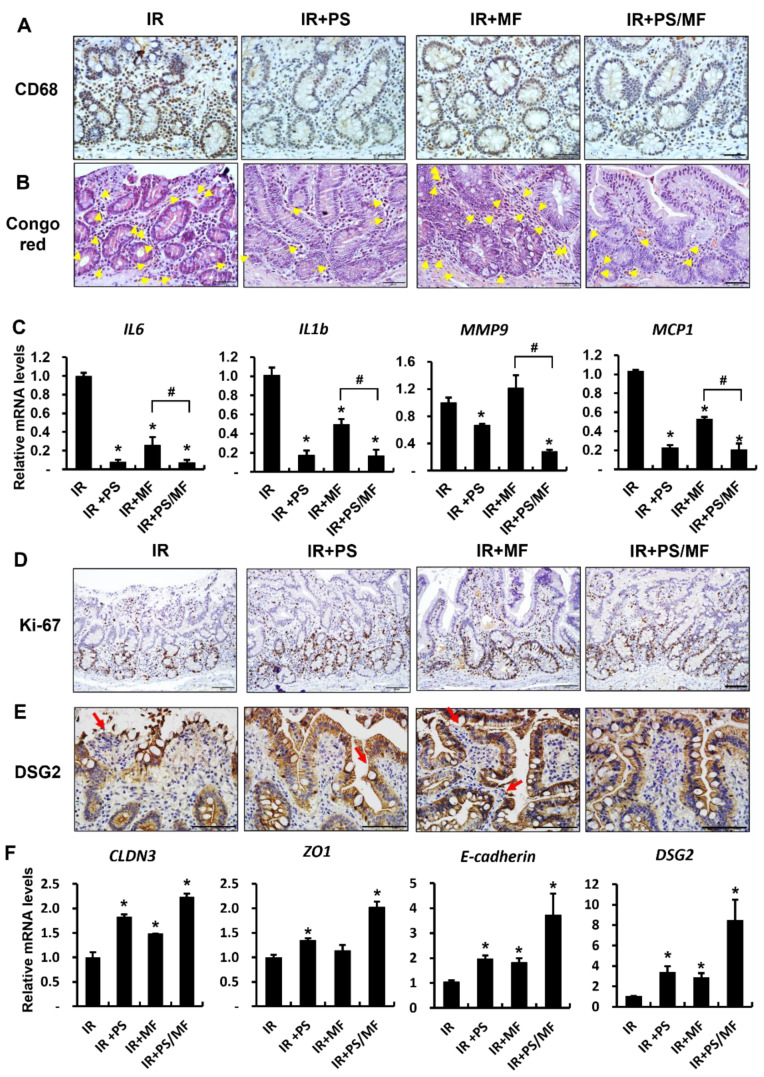
Effect of combined pravastatin and metformin treatment on inflammatory response and epithelial integrity in irradiated minipig model. (**A**) Immunohistochemical analysis of CD68 and (**B**) congo red stain in small intestine of irradiated (IR), pravastatin-treated IR (IR + PS), metformin-treated IR (IR + MF), combined PS and MF-treated IR (IR + PS/MF) minipigs. Yellow arrows indicate eosinophils. Bar = 50 μm. (**C**) mRNA levels of inflammatory cytokines, such as interleukin (IL) 6, IL1b, matrix metallopeptidase 9 (MMP9), and monocyte chemoattractant protein 1 (MCP1) in the small intestine of IR, IR+PS, IR+MF, IR+PS/MF groups. Immunostaining of (**D**) Ki-67 and (**E**) desmoglein 2 (DSG2) in the small intestine. Red arrows indicate defective DSG2 expression in the epithelium. Bar = 100 μm. (**F**) mRNA levels of intercellular junction molecules, such as claudin 3 (CLDN3), zonula occludens 1 (ZO1), E-cadherin, and DSG2 in the small intestine of IR, IR + PS, IR+MF, IR+PS/MF groups. Data are presented as the mean ± standard error of the mean; n = 4 per group. * *p* < 0.05 compared to the IR group; # *p* < 0.05 compared to the IR + MF group.

In our previous study on radiation-induced intestinal injury in a mouse model, leukocyte infiltration and production of inflammatory cytokines increased, resulting in an inflammatory response in the irradiated intestine. To classify inflammation occurring in the small intestine of minipig models, we performed immunohistochemical analysis of CD68 for monocytes and Congo red staining for eosinophils. As shown in Figure 6A and 6B, the number of CD68-positive cells and eosinophils in the IR group was significantly higher than that in the IR + PS, IR + MF, and IR + PS/MF groups (Figure 6A,B). IL6, IL1β, matrix metallopeptidase 9 (MMP9), and monocyte chemoattractant protein 1 (MCP1) are representative inflammatory cytokines in the irradiated intestine [12,33]. PS treatment or combined treatment with PS and MF significantly decreased the mRNA levels of IL6, IL1β, MMP9, and MCP1 compared with those in the IR group (Figure 6C). MF treatment also decreased the levels of these inflammatory cytokines, except MMP9 (Figure 6C). However, there were considerable differences in the expression of inflammatory cytokines between the IR + MF and IR + PS/MF groups. As the combined treatment of PS and MF affected epithelial proliferation in the mouse model, we also investigated epithelial proliferative ability in the minipig model. The expression of Ki-67 was higher in the IR + PS, IR + MF, and IR + PS/MF groups than in the IR group (Figure 6D). The mucosal barrier is composed of tight junction molecules (CLDN3 and ZO1), adherent junction molecules (E-cadherin), and desmosomes (DSG2) [29]. The expression of DSG2 protein was increased in the IR + PS, IR + MF, and IR + PS/MF groups compared with that in the IR group (Figure 6E). The mRNA levels of CLDN3, ZO1, E-cadherin, and DSG2 were notably higher in the IR + PS, IR + MF, and IR + PS/MF groups than in the IR group (Figure 6F). In particular, these junction molecules were most highly expressed in the IR + PS/MF group compared with the other groups (Figure 6E). These results suggest that the combined treatment of PS and MF in minipigs, as in mice, has a better effect on epithelial proliferation and epithelial junction molecules recovery than when used individually.

## 3. Discussion

As radiation-induced GI injury limits the survival of casualties from nuclear accidents or terrorism, GI damage is a critical factor responsible for mortality in humans and other mammals [38]. Radiation exposure of over 10 Gy mainly leads to GI injury with clinical symptoms, such as diarrhea, dehydration, sepsis, and intestinal bleeding, with eventual mortality within 10–15 days post exposure [39]. Furthermore, no Food and Drug Administration-approved agents are available to mitigate radiation-induced intestinal injury [40]. In this study, the combined treatment of PS and MF was more effective in alleviating radiation-induced intestinal injury than a single treatment. These data suggest that combined treatment of PS and MF may be used as a powerful therapeutic agent for radiation-induced enteropathy.

The anti-inflammatory effect of PS in radiation-induced GI injury has been demonstrated in mice and minipigs [10,12,13,14]. Recently, we reported that PS directly regulated epithelial barrier function and attenuated radiation-induced enteropathy in minipig models [14]. MF is a drug used worldwide for treatment of type 2 diabetes, and its various effects have been widely studied. The protective effects of MF on radiation-induced GI damage have been previously reported [17,19,20]. We also identified the stem cell preservative effects of MF in a radiation-induced intestinal injury mouse model [21]. However, a single therapy with PS or MF did not show survival effects in lethal dose irradiation in our data. To overcome this limitation, we used a combination of PS and MF for radiation-induced intestinal injury in mouse and minipig models in this study. We found that the combined treatment of PS and MF notably increased the survival rate compared with the single treatment or IR group in the lethal dose-irradiated mouse model. Histological analysis showed that the combined treatment of PS and MF dramatically attenuated histological damage, including villus length, crypt number, and histological score, compared with the other groups.

Furthermore, using minipig models, we investigated the therapeutic effects of combination treatment with PS and MF. Several in vivo experiments have been designed using rodents; however, there are limitations due to differences in anatomical, physiological, histopathological, and sensitivity to radiation between humans and rodents. We adjusted minipig models because not only is the GI function of minipigs similar to that of humans, but also the response to radiation exposure is highly similar to that of humans, which has been reported in several studies [41,42]. Therefore, minipigs are an appropriate animal model for the evaluation of pharmaceutical oral bioavailability following radiation damage. In the minipig model, combined treatment with PS and MF improved gross pathological damage in abdominal organs, including the stomach, intestine, and spleen, and attenuated radiation-induced intestinal histological damage.

Barrier damage with intercellular junction disruption is a well-known cause of radiation-induced intestinal injury [14,43]. Damaged intestinal epithelium increases permeability and promotes systemic influx of bacterial pathogens, resulting in inflammation and endotoxemia [34,44]. Clinical studies have shown that radiotherapy received patients exhibit increased intestinal permeability and tight junction disruption [43]. PS directly enhances intercellular junctions, including CLDN3, ZO1, and DSG2, in differentiated epithelial cells upon radiation exposure [14]. Tao et al. reported that adenosine monophosphate-activated kinase activation by MF accelerates tight junction molecules such as ZO1 and occludin and alleviates colitis [16]. In this study, while single treatment with MF did not markedly increase Cldn3 expression, single treatment with PS enhanced tight junction expression in the mouse model. In particular, the combined treatment of PS and MF markedly increased intercellular junction molecules, including CLDN3, ZO1, E-cadherin, and DSG2, compared with the single treatment groups in the mouse and minipig models. Therefore, combined treatment with PS and MF enhanced radiation-induced barrier damage by maintaining the effects of PS.

Radiation-induced intestinal injury results in inflammatory reaction with increased levels of inflammatory cytokines and chemokines, such as IL6, IL1β, MMP9, and MCP1 [12,14]. IL6 is an important mediator of the acute inflammatory phase [45]. IL1β, an inflammatory cytokine, is produced immediately following radiation exposure in epithelial and endothelial cells [46]. MMP9 is mainly produced by neutrophils and is the most abundantly expressed protease in inflamed tissues [47]. MCP1 is a chemokine that regulates migration and infiltration of monocytes and macrophages. Leukocyte infiltration in inflamed tissues is an important component of the progression of inflammatory processes. Neutrophils migrate into damaged tissue, generating a respiratory burst and contributing to the progression of the inflammatory response [48]. Eosinophils are pro-inflammatory leukocytes that comprise a small percentage of circulating blood cells [49]. Eosinophils also contribute to the inflammatory process by releasing various cytokines and chemokines, which are derived in concert with other inflammatory and immune cells during radiation exposure. PS has potent anti-inflammatory effects on radiation-induced intestinal injury [10,12,13]. Here, we also found that PS treatment showed anti-inflammatory effects, including inhibition of leukocyte infiltration and decreased inflammatory cytokine and chemokine expression in irradiated intestinal tissue. MF treatment did not inhibit the inflammatory response in the irradiated mouse model. In the mouse and minipig models, combined treatment with PS and MF effectively attenuated intestinal inflammation. Therefore, the combined treatment with PS and MF showed anti-inflammatory effects in radiation-induced intestinal injury by retaining the effects of PS.

The intestinal epithelium is one of the most rapidly proliferating tissues. Intestinal stem cells located in the crypt drive a processes of epithelium turnover and intestinal regeneration and generate precursors for specialized differentiated cells [50,51]. Therefore, these intestinal stem cells are reported to be responsible for intestinal regeneration after radiation damage [52]. The pathology of lethal GI tract damage is mainly involved in the depletion of the pool of intestinal stem cells, which impairs the regeneration of the villi-crypt structure and intestinal function [34,35]. MF was previously shown to mitigate radiation-induced enteropathy by promoting stem cell properties and epithelial proliferation in a mouse model and an ex vivo system [21]. In this study, we also identified that MF activated stem cell properties and alleviated radiation-induced intestinal injury in mice and minipig models. The combined treatment with PS and MF sustained the activated stem cell properties of MF in radiation-induced intestinal injury. Therefore, the combined treatment with PS and MF resulted in stem cell properties and epithelial regeneration in radiation-induced intestinal injury.

In conclusion, our findings highlight that the combination of PS and MF was more effective in alleviating radiation-induced enteropathy than a single treatment. Additionally, we suggest that minipigs may serve as an outstanding animal model for studying radiation-induced GI injury. Thus, the combined use of PS and MF is a promising therapeutic approach for treating radiation-induced enteropathy.

## 4. Material and Methods

### 4.1. Animal

#### 4.1.1. Mice

Male C57BL/6 mice (6-week-old) were obtained from DooYeol Biotech (Seocho-gu, Seoul, Republic of Korea) and sustained under specific pathogen free conditions at the animal facility of the Korea Institute of Radiological and Medical Sciences (KIRAMS). The mice were housed in a temperature-controlled room with a 12 h light/dark cycle, and food and water were provided ad libitum. The mice were acclimated for 1 week before the commencement of the experiments and were assigned to the following groups: (1) control, (2) irradiation (IR), (3) IR with PS treatment (IR + PS), (4) IR with MF treatment (IR + MF), and (5) IR with combined PS and MF treatment (IR + PS/MF). All animal experiments were approved by and performed in accordance with the guidelines of the Institutional Animal Care and Use Committee (IACUC) of KIRAMS (kirams 2021-0018).

#### 4.1.2. Minipigs

Eight male Gottingen minipigs (PWG Genetics Korea, PyungTek, Republic of Korea), weighing 20–25 kg each, were used in this study. The minipigs were physically examined for health before the experiments. They were housed indoors in individual cages and provided with dry pig food and filtered water. The minipigs were housed under environmentally controlled conditions at 22 ± 1 °C and 50 ± 10% relative humidity, with a 12 h light/dark cycle. The minipig care procedures were performed in accordance with the Guide for the Care and Use of Laboratory Animals of the Institute of Laboratory Animal Resources. All the animal experiments were approved by the IACUC of KIRAMS (kirams 2020-0090). Minipigs were randomized before treatment initiation in all experiments.

### 4.2. Irradiation and Treatment

#### 4.2.1. Irradiation and Treatment in Mice

The animals were anesthetized with an intraperitoneal injection of alfaxalone (85 mg/kg; Alfaxan^®^; Careside, Gyeonggi-do, Republic of Korea) and xylazine (10 mg/kg; Rompun^®^; Bayer Korea, Seoul, Republic of Korea). They were irradiated with a single exposure to 15 Gy or 13.5 Gy of whole abdominal lesion at a dose rate of 2 Gy/min using an X-RAD 320 X-ray irradiator (Softex, Gyeonggi-do, Republic of Korea). After exposure, the animals were orally administered 30 mg/kg/day PS (Prastan^®^; Yunjin Pharm, Seoul, Republic of Korea) and/or 500 mg/kg/day MF (Diabex, Daewoong Pharm. Co., Ltd., Seoul, Republic of Korea) for 30 d in the 15 Gy irradiated model and 6 d in the 13.5 Gy irradiated model.

#### 4.2.2. Irradiation and Treatment in Minipigs

The minipigs were anesthetized using an intramuscular injection of Zoletil 50 (4 mg/kg; Virbac, Republic of Korea) and xylazine (2.3 mg/kg; Rompun^®^; Bayer Korea, Seoul, Republic of Korea). The minipigs were randomly divided into four groups: IR, IR + PS, IR + MF, and IR + PS/MF. These minipigs were irradiated with 15 Gy at the abdominal lesions. For radiation exposure, minipigs were placed bilaterally in lateral recumbency. The mean dose rate at the center of the field was 1.12 Gy/min. The cranial landmark is located at the end of the xiphoid process. The irradiated field size was 20 × 30 cm under a 60 Co gamma-ray irradiation unit (Gamma Beam 100-80, 780; Best Theratronics, Ontario, Canada). The distance between the radiation source and spine was 80 cm. After irradiation, the minipigs were housed individually and provided dry food and filtered water. All minipigs were treated with a twice-daily oral dose of 40 mg/day PS (Prastan^®^; Yungin Pharm, Seoul, Republic of Korea) and/or 500 mg/day MF (Diabex, Daewoong Pharm. Co., Ltd., Republic of Korea) for 2 weeks. On days 13 and 14, the minipigs were sacrificed.

### 4.3. Histological Analysis of the Intestinal Tissue

Intestinal tissues of mice and minipigs were fixed with 10% neutral buffered formalin solution, embedded in paraffin wax, and sectioned transversely at a thickness of 4 µm. The slides were stained with H&E and Congo red. Histological scores were quantified in the H&E-stained slides and assessed by the degree of the epithelial maintenance, crypt damage, vascular dysfunction, and inflammation with infiltration of inflammatory cells. To investigate radiation-induced goblet cell damage, the slides were stained with PAS. For immunohistochemical analysis, the slides were subjected to antigen retrieval for 20 min and then treated with 0.3% hydrogen peroxide in methyl alcohol for 20 min to block endogenous peroxidase activity. After washing with PBS, the slides were blocked with 10% normal goat serum (Vector ABC Elite kit; Vector Laboratories, Burlingame, CA, USA) and incubated with primary antibodies, such as anti-Cldn3 (Invitrogen, Carlsbad, CA, USA), villin (Abcam, Cambridge, UK), Mpo (Abcam), ki-67 (Acris), Olfm4 (Invitrogen), and CD68 (Abcam) antibodies. Additionally, the slides were incubated with horseradish peroxidase-conjugated secondary antibody (Dako, Carpinteria, CA, USA) for 60 min. The peroxidase reaction was developed using diaminobenzidine substrate (Dako) prepared according to the manufacturer’s instructions, and the slides were counterstained with hematoxylin.

### 4.4. RNA Extraction, Reverse Transcription-Polymerase Chain Reaction (RT-PCR), and Real-Time PCR Quantification

Small intestine tissues of mice and minipigs were immediately snap-frozen and stored at −80 °C until RNA extraction was performed. Total RNA was isolated from the intestinal tissues using the TRIzol reagent (Invitrogen, Carlsbad, CA, USA) according to the manufacturer’s protocol. cDNA was synthesized using the AccuPower RT premix (Bioneer, Daejeon, Republic of Korea) according to the manufacturer’s protocol. Real-time RT-PCR was performed using a LightCycler 480 system (Roche, San Francisco, CA, USA). The mRNA expression levels of gene, determined using LightCycler 480 system software (Roche), were normalized to those of glyceraldehyde 3-phosphate dehydrogenase. Cycle threshold values were used to calculate the relative mRNA expression using the 2^−∆∆Ct^ method. The primer sequences are listed in Table 1.

### 4.5. Statistical Analysis

All quantitative data are expressed as the mean ± standard error of the mean. Statistical significance of differences was evaluated by performing Kaplan–Meier analysis followed by log-rank test for survival data and one-way analysis of variance with Tukey’s multiple comparison test for other data. Statistical significance was set at *p* < 0.05.

## Figures and Tables

**Table 1:** The primer sequences.

Species	Primer	Forward (5′–3′)	Reverse (5′–3′)
Mouse	*Cldn3*	AAGCCGAATGGACAAAGAA	CTGGCAAGTAGCTGCAGTG
	*Villin*	CACCTTTGGAAGCTTCTTCG	CTCTCGTTGCCTTGAACCTC
	*Il-1β*	GCAACTGTTCCTGAACTCA	CTCGGAGCCTGTAGTGCAG
	*Mcp-1*	GCAGTTAACGCCCCACTCA	CCCAGCCTACTCATTGGGATCA
	*Olfm4*	GCTGGAAGTGAAGGAGATGC	ACAGAAGGAGCGCTGATGTT
	*Lgr5*	TCAGTCAGCTGCTCCCGAAT	CGTTTCCCGCAAGACGTAAC
	*Gapdh*	AAGATGGTGATGGGCTTCCCG	TGGCAAAGTGGAGATTGTTGCC
Minipig	*IL-6*	TTCAGTCCAGTCGCCTTCT	GTGGCATCACCTTTGGCATCTTCTT
	*IL-1b*	ACCTGGACCTTGGTTCTC	GGATTCTTCATCGGCTTC
	*MMP-9*	AAGACGCAGAAGGTGGATTC	AACTCACACGCCAGAAGAAG
	*MCP-1*	TCTCCAGTCACCTGCTGCTA	AGGCTTCGGAGTTTGGTTTT
	*CLDN3*	GCCAAGATCCTCTACTCCGC	GAGAGCTGCCTAGCATCTGG
	*ZO1*	GAGGATGGTCACACCGTGGT	GGAGGATGCTGTTGTCTCGG
	*E-cadherin*	AAATGCTAGCTGGTGGGGAC	GCCTCCCATTGCTAACACCT
	*DSG2*	TCTTCCAGGCAGGGTCAAAC	CCAGGATCACAGTGCTTGGT
	*GAPDH*	GAAGGTCGGAGTGAACGGAT	CATGGGTAGAATCATACTGGAACA

## Data Availability

Not applicable.
